# Bedtime media use, psychological distress, and fatigue: a study of college students in Shaanxi Province, China

**DOI:** 10.3389/fpsyg.2025.1529137

**Published:** 2025-04-28

**Authors:** Ying Zhang, Xinfeng Cheng, Tolulope Ariyo, Wenjie Duan

**Affiliations:** ^1^School of Science, Xi’an Technological University, Xi’an, China; ^2^The Institute for Population and Management of Health Studies, Xi’an Technological University, Xi’an, China; ^3^School of Health Management, Shangluo University, Shangluo, China; ^4^Social and Public Administration School, East China University of Science and Technology, Shanghai, China

**Keywords:** psychological distress, fatigue, mental health, bedtime media use, visual stimulus, auditory stimulus

## Abstract

**Background:**

Numerous studies have linked psychological distress to fatigue, yet few have explored how bedtime media use mediates this relationship. This study examines whether using visual or auditory stimuli at bedtime mediates the relationship between psychological distress and fatigue among college students.

**Methods:**

A total of 1,831 Chinese college students (927 males and 904 females; mean age = 20.36 years, SD 1.26) from universities in Shaanxi Province, China, participated in the study. Data were collected using an electronic questionnaire that assessed psychological distress, bedtime media use, and fatigue. The bootstrap method was employed to test the mediating effects, with 5,000 random samples and a 95% confidence interval.

**Results:**

Psychological distress (*r* = 0.256, *p* < 0.001), visual stimuli of bedtime media use (*r* = 0.114, *p* < 0.001), and auditory stimuli of bedtime media use (*r* = 0.109, *p* < 0.005) were all positively related to fatigue. Among students with normal levels of psychological distress, the relationship between psychological distress and fatigue was partially mediated by the visual stimuli of bedtime media use. In contrast, for students with severe psychological distress, the auditory stimuli of bedtime media use mediated the relationship between psychological distress and fatigue.

**Conclusion:**

Based on the findings, psychological distress is indirectly associated with fatigue through the visual or auditory stimuli of bedtime media use. The visual and auditory stimuli exhibit different mediating effects among students with normal versus severe psychological distress. Interventions should focus on limiting bedtime media use to enhance health and reduce fatigue among college students experiencing psychological distress. Future studies may use longitudinal designs to establish causality or explore the reverse relationship between psychological distress and fatigue for a more robust finding.

## 1 Introduction

College life often comes with increased pressure in many aspects. An array of issues, ranging from social to economic or academic, may raise students’ stress levels, culminating in fatigue (Liu et al., 2023). Fatigue is a temporary state characterized by tiredness or prolonged exhaustion (Teuber et al., 2021), often caused by stress, overexertion, or lack of sleep (Lund et al., 2021). Inadequate sleep is particularly common among college students, with over 70% reporting insufficient sleep, making it a major contributor to fatigue (Mbous et al., 2022).

The easy access to electronic devices has made bedtime media use an important factor that affects sleep quality among college students. Research from Nagata et al. (2023) and Kortesoja et al. (2023) indicates that using digital media before bedtime leads to difficulties in falling asleep and overall decreases in sleep quality and duration, which in turn worsens fatigue symptoms. Additionally, psychological distress, which appears as stress along with anxiety and depression, pushes students to use bedtime media as a coping strategy, resulting in a cycle that reduces sleep quality and increases fatigue (Perveen et al., 2024).

Fatigue is especially common among students in China. Approximately two-thirds of Chinese high school students report experiencing moderate to severe fatigue, primarily due to academic pressure (Teuber et al., 2021). Additionally, over half of Chinese college students experience chronic fatigue linked to heavy workloads and sleep deprivation (Liu et al., 2023). In China, societal norms emphasizing academic achievement and familial expectations contribute significantly to adolescents’ stress, thereby heightening vulnerability to media addiction as a coping mechanism (Zhang et al., 2022). Furthermore, Chinese cultural values related to maintaining social harmony may limit open emotional expression, prompting adolescents to seek emotional outlets through intensive media engagement, thus intensifying psychological distress and fatigue (Cui et al., 2022).

Despite substantial research interest in fatigue due to its widespread prevalence and severe consequences, few studies have explored its modifiable lifestyle-related risk factors, such as bedtime media use, particularly among college students (Park et al., 2018; Malheiros et al., 2021). Additionally, existing fatigue research in China largely targets non-student populations or focuses on burnout rather than fatigue specifically, leaving the relationship between psychological distress and fatigue among Chinese college students under-examined (Chen et al., 2020; Liu et al., 2020, 2023; Gong et al., 2021). Therefore, investigating bedtime media use as a modifiable factor influencing the relationship between fatigue and psychological distress could provide essential insights and interventions to improve student health and well-being.

Psychological distress refers to a state of emotional suffering typically characterized by symptoms such as anxiety, depression, confusion, and a general feeling of being overwhelmed (Kop and Kupper, 2016; Sharp and Theiler, 2018; Collin et al., 2020). Research has consistently shown that psychological distress is positively related to fatigue (Shim et al., 2019; Collin et al., 2020; Yusoff et al., 2021; Estrella-Proaño et al., 2024). For instance, Estrella-Proaño et al. (2024) reported high fatigue, anxiety, and coping difficulties among first-year Ecuadorian undergraduates, primarily driven by deficient social support and family functionality.

The link between psychological distress and fatigue can be understood through physiological and behavioral mechanisms. When the body responds to stress, cortisol—a stress hormone, is released. This stress hormone has the capability to cause physical and mental depletion, causing psychological distress and fatigue (Gecaite-Stonciene et al., 2022). Additionally, stress can cause insomnia, preventing proper mental and physical recovery and contributing to fatigue (Van Someren, 2021). Although this interplay can exist in a loop, other factors, such as bedtime habits, may exacerbate how psychological distress impacts fatigue.

While the link between psychological distress and fatigue is well-established, the potential mediating effects of media use are less examined. A review of studies between 2010 and 2020 indicated that adolescents’ poor emotional regulation leads to addictive behaviors, including increased media use (Gioia et al., 2021b). Recent research further indicates that excessive media consumption, particularly at bedtime, can exacerbate sleep disturbances and contribute to fatigue. For instance, a Norwegian study found that each hour of screen time in bed was associated with 24 fewer minutes of sleep and a 59% higher risk of insomnia, highlighting the negative impact of bedtime media use on sleep quality and duration (Hjetland et al., 2025). Studies also demonstrate that psychological distress may result in heightened media use as a coping strategy, thereby impairing sleep quality and exacerbating exhaustion (Gioia et al., 2021a).

Several theories explain how bedtime media use may influence the relationship between psychological distress and fatigue. According to the uses and gratifications theory, people actively seek and use media to satisfy their needs, which may be based on various factors, including psychological, social, information, or entertainment (Rubin, 2009). For people experiencing psychological distress, bedtime media use may be a sort of coping mechanism. Furthermore, the mood management theory suggests that media users select content based on their current emotional state, following the principle of mood optimization (Reinecke, 2016). In other words, individuals intentionally select media types (visual or auditory) that they believe will enhance or regulate their mood.

Numerous studies have linked bedtime media use to morning fatigue, attributing it to increased sensory stimulation that disrupts sleep patterns and circadian rhythms (Orzech et al., 2016; Lastella et al., 2020; Tam et al., 2021). While some evidence indicates both visual and auditory stimuli from nighttime media exposure cause fatigue (Liu et al., 2023; Saling and Haire, 2016), other studies suggest only visual stimuli have significant effects (Guan and Duan, 2020). Thus, further research is needed to clarify these findings.

**Figure 1:**
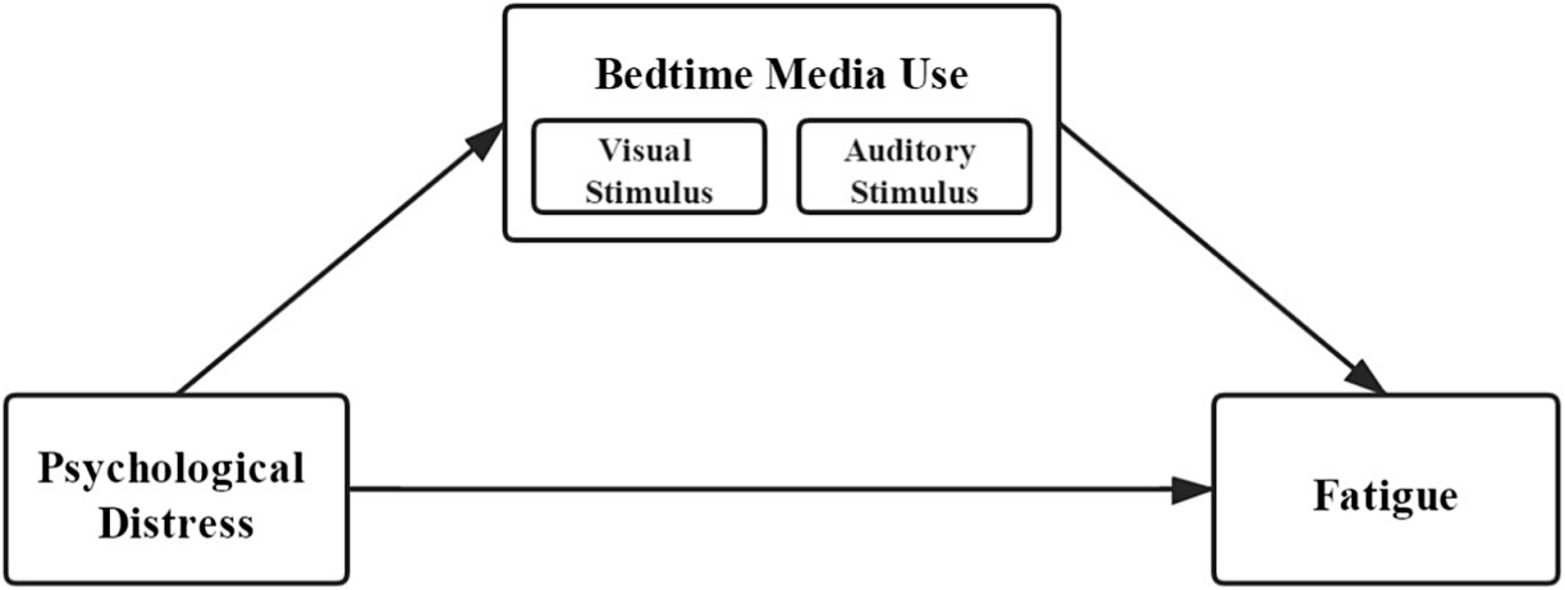
Model of the mediation effect of the stimulus of bedtime media uses between psychological distress and fatigue.

In this study, we intend to explore the mediating role of bedtime media use in the relationship between psychological distress and fatigue among Chinese college students. Two objectives are involved. First, we sought to confirm if the positive association between psychological distress and fatigue reported in previous studies holds true for the current study sample. Second, we seek to extend the existing literature by examining the mediating role of bedtime media use—specifically visual and auditory stimuli—in this relationship. Visual and auditory stimuli were selected as mediators because previous research indicates that these sensory stimuli during bedtime media use are most directly linked to sleep disruption and increased cognitive arousal, thereby potentially exacerbating fatigue and psychological distress. While other forms of media interaction (e.g., text-based media or social media interactions without audiovisual elements) could also have potential effects, they were excluded to focus explicitly on sensory-driven factors that have more direct neurophysiological implications for sleep quality. The theoretical model is shown in Figure 1, depicting how bedtime media use (categorized into visual and auditory stimuli) mediates the relationship between psychological distress and fatigue, in addition to a direct effect of psychological distress on fatigue. Findings from this study will be relevant in understanding and providing interventions to improve the well-being of college students in China.

## 2 Materials and methods

### 2.1 Data and participants

The data analyzed in this study were obtained from an electronic questionnaire survey conducted in Shaanxi Province, China, in October 2021. The participants in the survey were university freshmen who had daily access to a media device. A power analysis was conducted using G*Power software (Mayr et al., 2007) prior to data collection to determine the minimum required sample size. Based on an anticipated medium effect size (f^2^ = 0.15), a significance level of *α* = 0.05, and a desired statistical power of 0.95, the analysis indicated a minimum sample size requirement of 138 participants. The questionnaire was administered to 1875 respondents; however, 2.4% (44 respondents) did not complete the questionnaire fully, resulting in a final analytical sample of 1831 respondents. This sample size substantially exceeds the minimum requirement, ensuring robust statistical power for analysis.

Ethical approval for the study was obtained from the Human Research Ethics Committee of the Department of Sociology, Xi’an Technological University, China, and the research adhered to the Declaration of Helsinki. Written informed consent was obtained from all participants.

### 2.2 Measures

The dependent variable in this study is fatigue. This variable was measured using the Chinese translation of the Patient-Reported Outcomes Measurement Information System (PROMIS) Fatigue short form 7a (Yang et al., 2019), tested and validated for the Chinese population (Cook et al., 2016). The scale measures general fatigue, encompassing both physical and mental fatigue. It is a self-reported 7-item scale, consisting of items such as, “How often did you experience extreme exhaustion in the past 7 days?” Each item was measured on a 5-point scale ranging from 1 = never to 5 = always. The Cronbach’s alpha coefficient was 0.78, indicating internal consistency. For analytical purposes, the scores were transformed into a standardized T-score.

The main independent variable in this study is psychological distress, measured using the short version of the Depression Anxiety Stress Scale (DASS-21), a validated self-report tool assessing general psychological distress (Henry and Crawford, 2005). The DASS-21 comprises half the items of the original DASS-42 and is particularly suitable for population-level research, while the full version (DASS-42) is more oriented toward clinical settings and endocrine indicators (Antony et al., 1998). The DASS-21 evaluates three dimensions of psychological well-being: depression, anxiety, and stress. Respondents rated their experiences over the past week (e.g., “I felt that life was meaningless”) using a four-point scale ranging from 0 (“This item does not apply to me at all”) to 3 (“This item applied to me very much”), with no items reverse-coded. The scores were aggregated to generate the psychological distress variable. The scale has demonstrated good validity in the Chinese context (Wang et al., 2016), and internal consistency was strong in this sample (Cronbach’s alpha = 0.89). Given its focus on general psychological distress rather than specific conditions, the DASS-21 is suitable for exploratory research. Additional independent variables included demographic characteristics such as sex (male = 1, female = 0) and age, which was coded as a continuous variable.

The mediating variable in this study is bedtime media use, divided into two categories: visual stimuli (e.g., playing computer games, watching videos, internet browsing, texting, and reading) and auditory stimuli (listening to music and making phone calls). Using a six-point Likert scale (1 = never to 6 = always), participants reported how frequently they used each media activity to help them fall asleep. For analysis, responses for each category were averaged separately to derive scores for visual and auditory bedtime media use.

### 2.3 Data analysis

Three levels of analysis were performed in this study. First, we conducted a descriptive analysis using a correlation matrix to determine the associations between psychological distress, stimulus of bedtime media use, and fatigue. Secondly, multiple linear regression analyses were conducted to examine the roles of psychological distress and bedtime media use as predictors of fatigue. Three models were fitted; the first included the demographic factors—sex (male = 1, female = 0) and age. Psychological distress was added in the second model, while both variables of bedtime use stimuli were added in the third model.

Third, we utilized the structural equation model (SEM) to conduct a mediation analysis. We examined whether bedtime media use stimuli mediated the relationship between psychological distress and fatigue. This analysis was performed across two groups—normally and severely psychologically-distressed groups. Bootstrapping was conducted with 5,000 samples to provide robust estimates of the 95% confidence intervals (CIs) for the standardized effects.

In the SEM analysis, psychological distress (*X*) is the predictor, fatigue (*Y*) is the outcome variable, and the visual stimulus of bedtime media use (*M_1_*) and auditory stimulus of bedtime media use (*M_2_*) were the mediators. In this framework, path a_1_ illustrates the direct influence of psychological distress on the visual components of bedtime media consumption. Path a_2_ depicts the direct effect of psychological distress on the auditory elements of bedtime media use. Likewise, path b_1_ represents the direct impact of visual bedtime media stimuli on fatigue, while path b_2_ signifies the direct effect of auditory bedtime media stimuli on fatigue. Path c denotes the direct relationship between psychological distress and fatigue. The mediating effect, represented by path c′, captures how psychological distress affects fatigue through both visual and auditory bedtime media stimuli, calculated as c′ = (a_1_ × b_1_) + (a_2_ × b_2_). All *p*-values are two-tailed, and the level of statistical significance was set at *p* < 0.05. All analyses are conducted using STATA 15.0 software.

## 3 Results

### 3.1 Participation and sample characteristics

A total of 1831 samples were included for analysis. The mean age of participants was 20.36 years (SD = 1.26, range = 17–25), and nearly 50% (904 participants) were female. The main independent variable—psychological distress, has a mean score of 14.225 (SD = 12.320, range = 0–63). Based on this score, the participants were categorized into three groups: normal psychologically distressed students (cutoff = 0 to 20), moderate psychologically distressed students (cutoff = 21 to 31), and severe psychologically distressed students (cutoff = 32 to 63). The majority of the respondents (72.3%) fall under the first group, while about 9.4% fall under the third group. The mean score of the standardized T-score of the fatigue scale is 50 (SD = 10).

### 3.2 Bivariate correlation analyses

Table 1 reports the results of correlation analyses between variables representing stimuli of bedtime media use—visual and auditory stimuli, psychological distress, and fatigue. The Table shows that psychological distress was positively associated with fatigue (*r* = 0.69, *p* < 0.05). Additionally, psychological distress showed a significant positive association with the visual stimulus of bedtime media use (*r* = 0.35, *p* < 0.05), while its association with the auditory stimulus of bedtime media use was not significant (*r* = 0.207, *p* > 0.05). The visual stimulus of bedtime media use was positively related to fatigue (*r* = 0.370, *p* < 0.05), and the relationship between auditory stimulus of bedtime media use and fatigue was also significant (*r* = 0.627, *p* < 0.05). These findings suggest that psychological distress may influence visual and auditory bedtime media use stimuli differently. Furthermore, the two types of bedtime media use stimuli may have distinct effects on fatigue.

**Table 1:** Correlation analysis of psychological distress, stimulus of bedtime media use and fatigue.

	1	2	3	4
1	–			
2	0.63^a^	–		
3	0.35^a^	0.21	–	
4	0.37^a^	0.26	0.69^a^	–

1 = Visual stimulus of bedtime media use, 2 = Auditory stimulus of bedtime media use, 3 = Psychological distress, 4 = Fatigue.

a *p < 0.05.*

### 3.3 Multiple linear regression analysis

The results of the multiple linear regression are presented in Table 2. Three models were fitted, as previously stated in the methods section. While sex did not have a statistically significant relationship with fatigue in Model 1, age demonstrated a significant positive association (*β* = 0.42; *t* = 4.22; *p* < 0.001). In Model 2, psychological distress was positively related to fatigue (β = 0.28; *t* = 40.75; *p* < 0.001). The two types of bedtime media stimuli—visual and auditory, were entered in Model 3, and the results showed that both had a small yet significant explained variance to fatigue. Visual stimulus was (β = 0.11; t = 5.76, *p* < 0.001), while auditory stimulus was (β = 0.11; *t* = 2.06, *p* < 0.05). The corresponding F-statistics indicated that the models were statistically significant.

**Table 2:** The results of multiple linear regressions of psychological distress and stimulus of bedtime media use on fatigue.

	Model 1			Model 2			Model 3		
	*b*	*t*	*p* value	*b*	*t*	*P* value	*b*	*t*	*p* value
Constant	7.95	4.51	0.000	5.60	4.10	0.001	3.42	2.51	0.012
Sex	0.35	1.50	0.135	0.57	3.37	0.047	0.66	3.99	<0.001
Age	0.42	4.22	0.000	0.33	4.96	0.49	0.33	5.05	<0.001
Psychological distress				0.28	40.75	0.001	0.26	36.04	<0.001
Visual stimulus							0.11	5.76	<0.001
Auditory stimulus							0.11	2.06	0.039
*R^2^*	0.01			0.48			0.50		
*F*	11.31			567.84			372.12		
*P* value	0.000			0.000			0.000		

### 3.4 Mediation analyses

#### 3.4.1 Normal level of psychological distress

**Figure 2:**
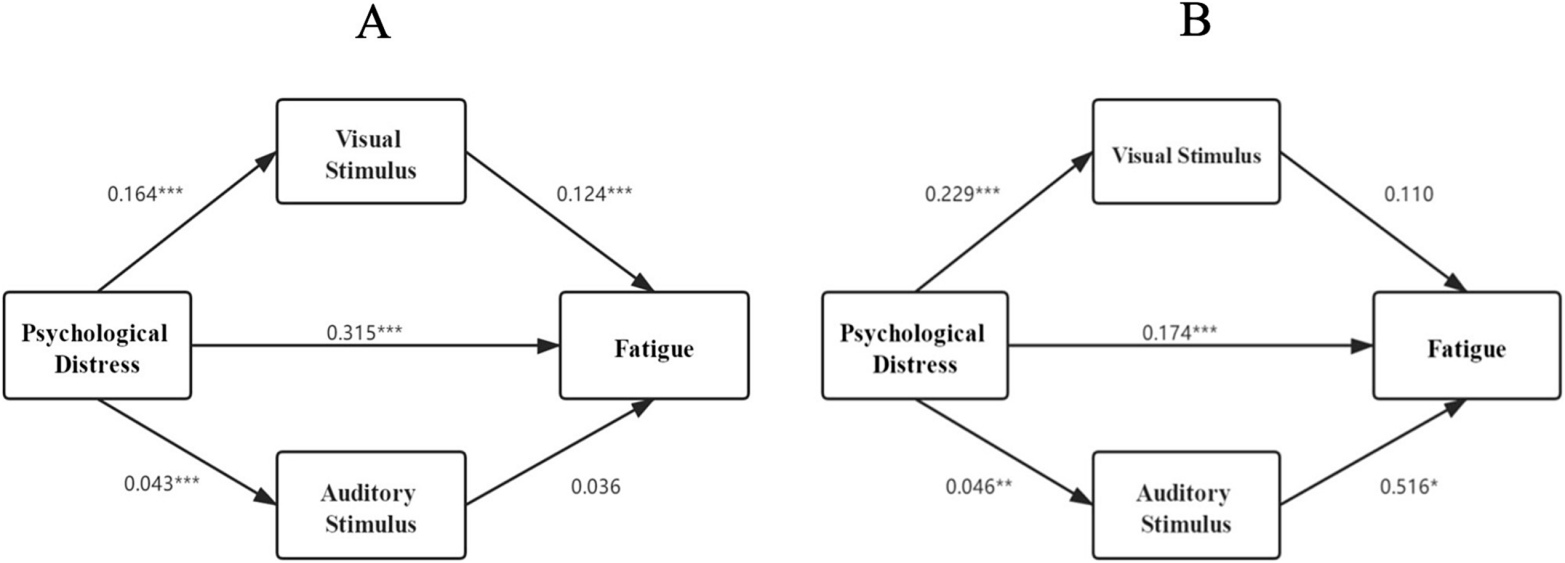
The SEM of the association between psychological distress, stimuli of bedtime use, and fatigue. **(A)** normal psychologically distressed; **(B)** severely psychologically distressed.

In the first mediation analysis, the sample was restricted to students with normal levels of psychological distress (*N* = 1,324), and the role of the stimulus of bedtime media use (visual and auditory stimuli) was investigated in the relationship between psychological distress and fatigue. The result of the SEM is shown in Figure 2A. It indicates that psychological distress is significantly related to visual stimulus of bedtime media use (*β* = 0.164, *p* < 0.001), and visual stimulus of bedtime media use is significantly related to fatigue (β = 0.124, *p* < 0.001).

Furthermore, Table 3 presents the quantified total effect size of the mediation paths. Consistent with the results of the bivariate analysis, psychological distress remained associated with fatigue when controlling for bedtime media use. Psychological distress exerted an indirect effect on fatigue through the visual stimuli of bedtime media use. This finding suggests that the relationship between psychological distress and fatigue is partially mediated by the visual stimuli of bedtime media among college students with typical levels of psychological distress.

**Table 3:** The mediation effect sizes of stimulus in the relationship between psychological distress and fatigue.

Level of psychological distress	Path	Effect type	Effect size	*p*-Value
Normal	Psychological distress— > Fatigue	Direct effect	0.315	<0.001
		Indirect effect	0.022	<0.001
Severe	Psychological distress— > Fatigue	Direct effect	0.174	<0.001
		Indirect effect	0.049	0.007

#### 3.4.2 Severe level of psychological distress

In the second mediation analysis, the sample was restricted to students with severe levels of psychological distress (*N* = 173), and the role of the stimulus of bedtime media use (visual and auditory stimuli) was investigated in the relationship between psychological distress and fatigue.

The SEM is shown in Figure 2B, indicating that psychological distress is significantly related to the auditory stimulus of bedtime media use (β = 0.046, *p* = 0.007), and visual stimulus of bedtime media use was significantly correlated to fatigue (β = 0.516, *p* = 0.015).

Furthermore, Table 3 shows the total effect size of the mediation paths. Consistent with the results of the bivariate analysis, psychological distress remained associated with fatigue even when controlling for bedtime media use. This finding suggests that among college students experiencing severe levels of psychological distress, the relationship between psychological distress and fatigue is partially mediated by the auditory stimuli of bedtime media.

## 4 Discussion

To explore the mechanisms linking psychological distress and fatigue, specifically the mediating role of bedtime media use, we analyzed primary data collected through an electronic questionnaire in October 2021. Three levels of analysis were conducted: bivariate correlation analysis, multiple regression analysis, and mediation analysis using structural equation modeling (SEM). The findings indicate that (1) there is a strong positive relationship between psychological distress and fatigue, suggesting that higher psychological distress is associated with greater fatigue levels among students. (2) In students with normal psychological distress, visual stimuli from bedtime media use partially mediated the link between distress and fatigue. In contrast, for students with severe psychological distress, auditory stimuli played a more significant mediating role.

Overall, the study extends the body of knowledge, showing that bedtime media stimuli differentially mediate the effect of psychological distress on fatigue, with the type of stimulus (visual or auditory) varying based on distress levels. This knowledge can be used to optimize sleep hygiene through specific recommendations appropriate to an individual’s levels of psychological distress. For instance, individuals with higher distress might be better off avoiding viewing screens before bed and instead engaging in calming auditory stimulation. These individual-oriented treatments can help improve sleep quality and decrease fatigue to boost general well-being.

First, we found a significant positive association between psychological distress and fatigue, which is consistent with previous studies across various contexts, including Asia (Shim et al., 2019; Yusoff et al., 2021), Australia, and Europe (Collin et al., 2020). This effect may be attributed to how psychological distress hinders a person’s ability to cope with stressors, depleting mental resilience and intensifying feelings of fatigue (Liu et al., 2017; Zhang et al., 2022). Among Chinese students, academic pressure and the desire to meet social expectations may heighten stress levels (Li et al., 2017; Zhang et al., 2022), intensify psychological distress, and increase fatigue.

Furthermore, the results indicated that visual and auditory bedtime stimuli mediate the relationship between psychological distress and fatigue based on the levels of distress. Students experiencing psychological distress are more likely to use media stimuli for emotional regulation, particularly during bedtime (Orzech et al., 2016; Gioia et al., 2021b). However, using media before bed can affect the endocrine system, disrupt circadian rhythms, and interfere with sleep quality (Orzech et al., 2016; Tam et al., 2021). Since students’ schedules are constrained by school hours, insufficient nighttime sleep due to media use can lead to fatigue the following day (Tam et al., 2021).

The different mediating effects of visual and auditory stimuli on the relationship between psychological distress and fatigue reflect how each interacts with cognitive and physiological processes. Visual stimuli, like light from screens, disrupt circadian rhythms and delay melatonin production, leading to sleep difficulties and fatigue (Chellappa, 2021). Individuals with typical psychological distress may manage stress with moderate nighttime media use, but excessive visual media before bed can still delay sleep onset and result in morning fatigue.

For those with severe distress, auditory stimuli—such as media sounds before bed—have a stronger effect on fatigue by provoking heightened arousal and rumination, which interfere with relaxation and sleep quality (Pacheco-Unguetti and Parmentier, 2014). This suggests that while visual stimuli subtly contribute to fatigue through sleep disruption, auditory stimuli more directly exacerbate fatigue in highly distressed individuals by intensifying cognitive and emotional arousal before sleep.

This study highlights important policy and practice implications for addressing fatigue and psychological distress among students through tailored mental health interventions, curriculum adaptation, awareness campaigns, and technological solutions. Given that visual and auditory stimuli from bedtime media use mediate fatigue differently based on psychological distress severity, policymakers and educators should develop specific guidelines. For students experiencing normal distress, policies should target limiting visual media use before bedtime; for those with severe distress, restrictions on auditory stimuli may be more beneficial. Schools should offer regular mental health screenings and accessible counseling to identify issues early and provide personalized fatigue and sleep management strategies. Incorporating mental health, stress management, and healthy media use education into curricula, supported by community awareness campaigns, can further improve student well-being. Technological solutions, such as screen-time control apps, should be promoted to help students manage media exposure, thereby reducing fatigue and enhancing overall health.

This study has several limitations. First, the use of cross-sectional data limits our ability to draw causal conclusions. Second, although psychological distress and fatigue may interact in a bidirectional relationship, we did not examine the potential reverse association in this study. Third, due to data limitations, we could not incorporate variables related to family background, even though family factors significantly influence individuals’ mental and emotional development. Additionally, daytime physical activity, another factor not measured in this study, could independently affect fatigue levels, possibly resulting in omitted variable bias when assessing the impact of bedtime media use. Lastly, although videos generally contain both visual and auditory stimuli, our analysis categorized videos primarily as visual stimuli following established literature practices. Future research should clearly differentiate between visual, auditory, and combined media stimuli and control for family background and physical activity factors to provide a more robust understanding of their distinct effects on fatigue. Furthermore, exploring the reverse association between psychological distress and fatigue using longitudinal or experimental designs could help clarify potential bidirectional relationships and inform more effective interventions.

## 5 Conclusion

This study examined the mechanisms of association between psychological distress and fatigue, particularly the mediating role of bedtime media use. The data obtained through an electronic questionnaire in 2021 were analyzed using bivariate correlation analysis, multiple regression analysis, and mediation analysis. While the finding validates the positive association between psychological distress and fatigue among Chinese college students, it also showed the association is mediated by visual or auditory bedtime media use based on distress levels. Recommendations for policy and practice cover multiple dimensions, including mental health support, curriculum adaptation, awareness campaigns, and technological solutions to improve sleep hygiene, reduce fatigue, and ultimately improve students’ overall well-being. Future studies should employ longitudinal designs to establish causality, explore the reverse association between psychological distress and fatigue, and account for household characteristics for a more robust analysis.

## Data Availability

The raw data supporting the conclusions of this article will be made available by the authors, without undue reservation.
